# Reaching gender parity and improving internationalisation after five decades of FSBI symposia, but subtle career‐stage effects on timekeeping remain

**DOI:** 10.1111/jfb.70449

**Published:** 2026-04-07

**Authors:** Joshka Kaufmann, William Bernard Perry

**Affiliations:** ^1^ Marine Institute Newport Ireland; ^2^ Water Research Institute, School of Biosciences Cardiff University Cardiff United Kingdom

**Keywords:** academic equity, career stage, conference behaviour, fisheries science, gender bias, internationalism, representation, timekeeping

## Abstract

We analysed 57 years of Fisheries Society of the British Isles (FSBI) symposia, showing speakers with traditionally female names increased from near absence to reaching parity in recent symposia. International affiliations increased in the late 1980s, peaking in 2024 with 27 countries, albeit with overrepresentation of high‐income countries. Timekeeping for 68 presentations at the 2025 symposium showed that senior scientists were more likely to overrun than junior presenters; no systematic gender differences were detected. Although gender representation has improved, timekeeping asymmetries reveal career‐stage effects, underscoring the need for consistent data collection, moderation and equitable session management.

Biases in visibility at scientific conferences have received increasing scrutiny across disciplines. Across life sciences, studies generally report that women, racial and ethnic minorities and early‐career researchers are underrepresented among speakers (Falk & Hagsten, 2024; Klein et al., 2017; Le et al., 2021; Schroeder et al., 2013; Wiley et al., 2024), particularly for high‐visibility presentations (Isbell et al., 2012). For example, at an international marine science conference, junior female scientists were disproportionately assigned – both by conveners and through self‐selection (Jones et al., 2014) – to poster presentations rather than oral talks, compared to male colleagues and more senior researchers (Johannesen et al., 2023). In addition, women tend to participate less frequently during postpresentation discussions, asking fewer questions overall (Hinsley et al., 2017; Jarvis et al., 2022; Käfer et al., 2018).

Such disparity can reinforce cumulative career advantages through visibility and networking and may reflect (subtle, unconscious) biases in how authority or entitlement to speak are expressed and perceived (Llorens et al., 2021). Despite slow but notable improvements in representation of previously underrepresented groups (Käfer et al., 2018), behavioural asymmetries still occur today. Research on gender imbalances in fisheries and aquatic sciences is still developing, with some initial evidence available (Johannesen et al., 2023). Our objectives were to (1) assess temporal trends in gender and geographical representation at Fisheries Society of the British Isles (FSBI) symposia over five decades and (2) evaluate differences in timekeeping by gender and career stage at the joint FSBI–Institute of Fisheries Management (IFM) 2025 symposium.

Holding its inaugural meeting at the Zoological Society of London on 21 October 1967, the FSBI has grown into an international, non‐political, learned society that supports scientific activity in fish biology and fisheries science through charitable activities. It held its first official annual symposium in 1977 (although pre‐1977 scientific meetings are also referred to as symposia in this manuscript for simplicity), with the most recent 2025 symposium taking place in Belfast, United Kingdom. This most recent symposium was the first time the FSBI had held a joint annual symposium with their partner organisation, IFM – a partnership which began in December 2020. The IFM is another international organisation based in the United Kingdom, founded in 1969, dedicated to the advancement of sustainable fisheries management. The FSBI has long been committed to supporting equity, diversity and inclusion (EDI), especially of early‐career researchers, with a formal recognition of the Society's commitment in 2022 with the creation of an EDI Committee and corresponding EDI statement (https://fsbi.org.uk/edi).

We quantified gender representation and country of affiliation among invited and contributed speakers across 57 years of FSBI symposia (1968–2025). Speaker gender was determined from first names using historical databases, with the R package *gender* (Blevins & Mullen, 2015) and genderize.io for contemporary and international names (Sebo, 2021).

A total of 1396 speakers were included across 25 symposia; gender could not be confidently assigned for 40 individuals (2.9%). Where abstract books listed only first initials (*n* = 272, 20%), first names were retrieved through targeted online searches (e.g., ResearchGate, institutional webpages, obituaries). Career stage was not included, as largely unknown, but we separated invited speakers (*n* = 100, 7.2%) from regular presenters. Rigorously assessing underrepresentation along axes other than gender is both challenging and ethically sensitive. Information on institutional power, disability status and ethnic or cultural background of symposia attendees assessed here was unavailable, incomplete or inappropriate to infer. Similarly, gender inference based on first names is far from a perfect proxy, as gender alone is a complex and multifaceted identity that cannot be fully captured through names alone, but it is used here out of necessity.

The proportion of women speakers at FSBI symposia increased markedly over five decades, rising from near absence in the 1970s to only just reaching parity in recent symposia (Figure 1a). This trend was evident for both invited and non‐invited presentations. Invited speakers appeared to be slightly male‐biased. Relative to contributed speakers, they consistently showed lower gender parity, alongside greater inter‐annual variability, likely reflecting small sample sizes. Across years, invited speakers showed a small but consistent difference in female representation relative to non‐invited speakers [−5%, 95% confidence interval (CI): −11% to 0.8%], despite substantial year‐to‐year variability. These patterns suggest that although long‐term representation has improved, parity during the selection of invited speakers could be improved to reach consistent gender visibility across all presentation formats. Our results support the encouraging trends indicating that gender parity among fisheries and aquatic scientists presenting at conferences is being reached (Bombaci et al., 2025; Käfer et al., 2018; Johannesen et al., 2023). These results likely reflect improvements in the community itself but also conscious efforts from conference organising committees and learned societies. However, it is important to note that disparity between women and men in senior roles, leadership positions, grant acquisition and scholarly publishing remains (Kozlowski et al., 2022; Larivière et al., 2013).

**FIGURE 1:**
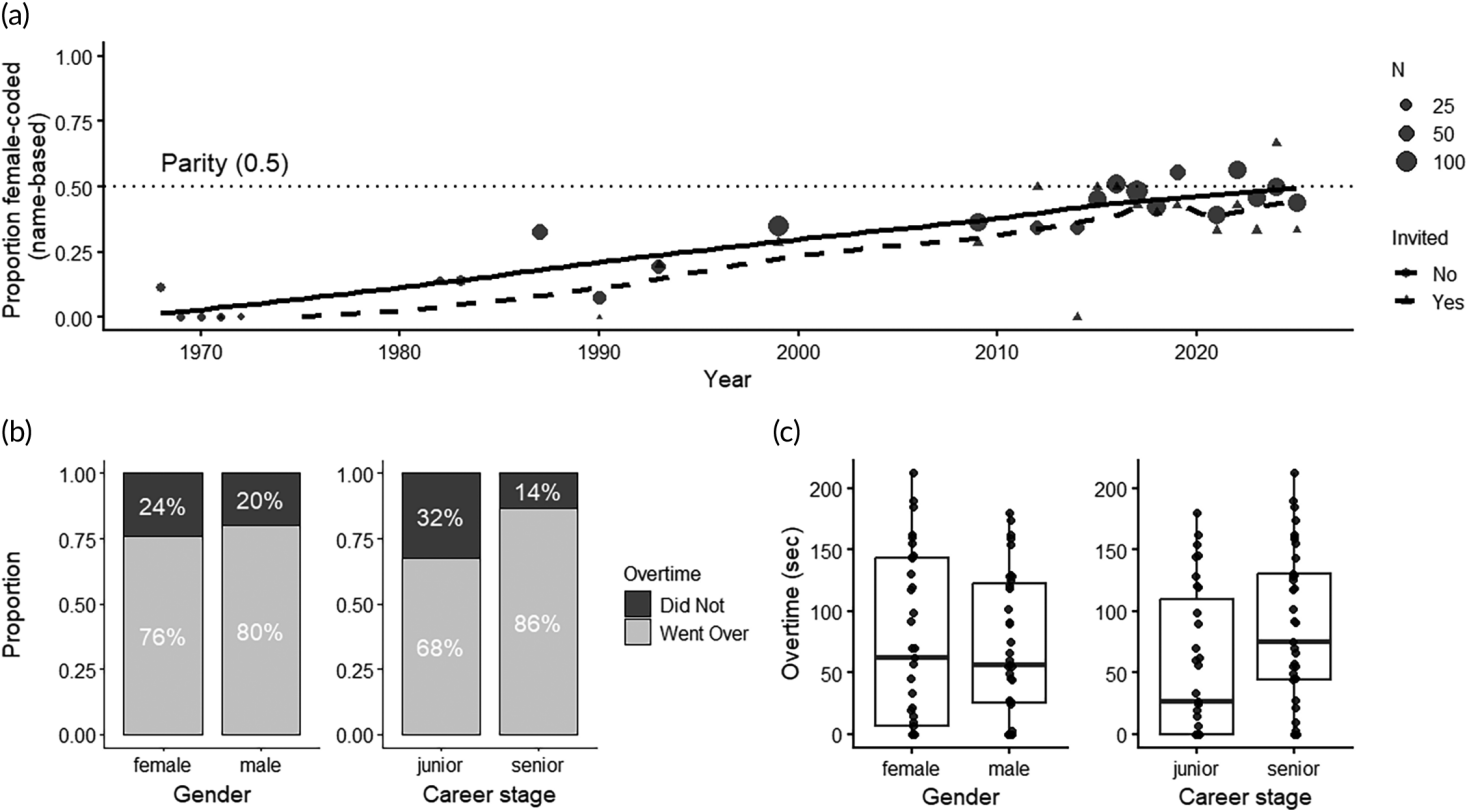
Gender representation and overtime patterns across career stages and years. (a) Proportion of female‐coded individuals (based on name) over time, with points sized by sample size (*N*) and lines indicating whether individuals were invited (circles and solid line) or not (triangles and dashed line). The horizontal dotted line indicates gender parity (0.5). (b) Proportion of individuals who went over allotted time by gender or career stage. Percentages within bars indicate the proportion that did (light grey) or did not (dark grey) exceed time. (c) Distribution of overtime duration (seconds) by gender and career change.

Before 1987, only six countries from Europe and the Americas participated. Since then, variation in geographic affiliation of participants has grown steadily, including nations from Africa, Asia and Oceania, reaching 27 countries in 2024 (Figure 2a). Participation was heavily concentrated in a small number of countries, with the United Kingdom understandably dominating, given the history of the Society (Figure 2b,c). The United States and Canada followed, whereas all other countries contributed comparatively modest numbers. Consistent with this pattern, the majority of participants originated from high‐income economies, with minimal representation from upper middle‐, lower middle‐ and low‐income economies (Figure 2d), based on the World Bank income classification for the specific symposium year (https://datacatalog.worldbank.org/).

**FIGURE 2:**
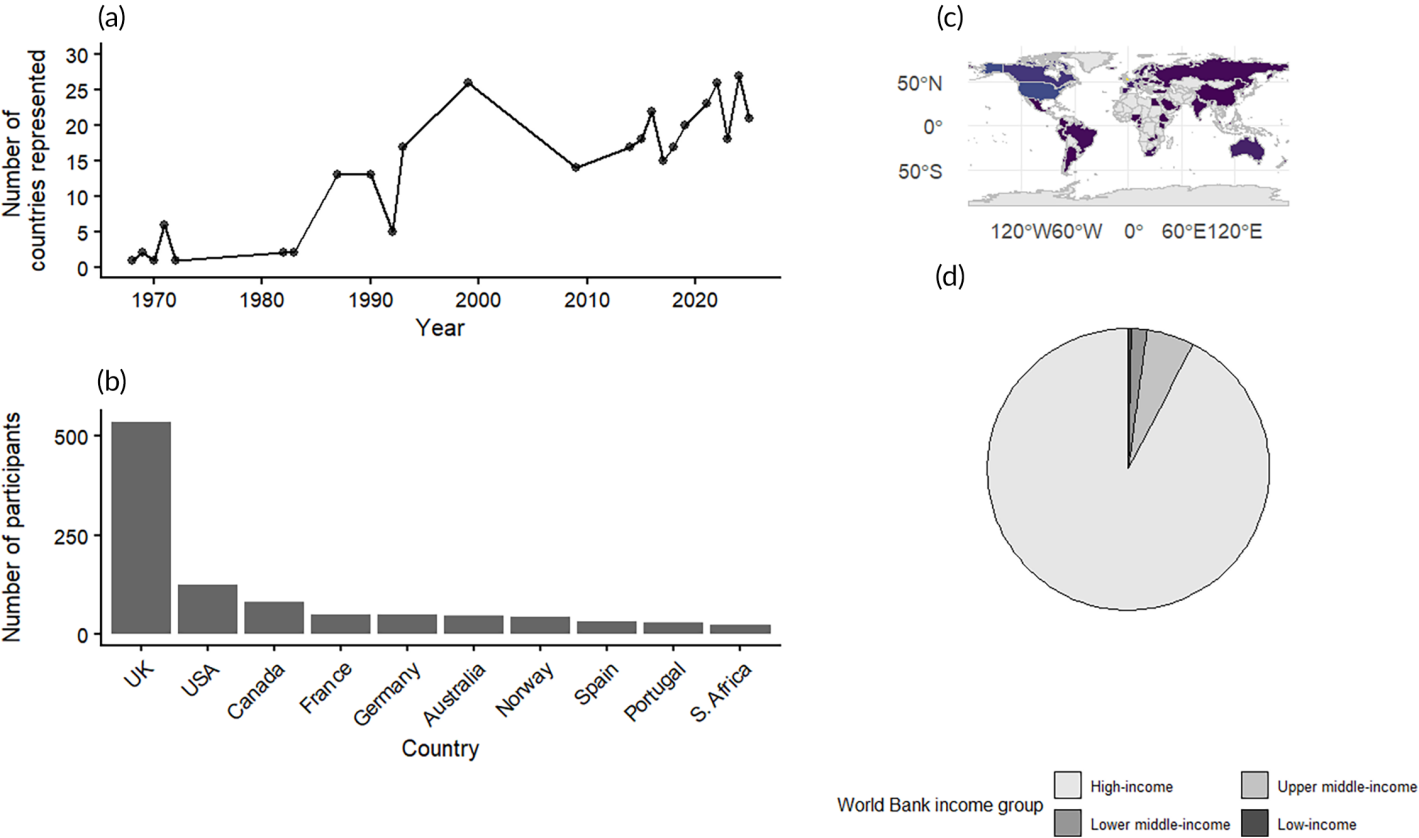
Geographical affiliation of Fisheries Society of the British Isles (FSBI) presenters across years. (a) Number of countries represented at FSBI symposiums over time. (b) Number of total participants for the top 10 countries. (c) Map showing representative countries. (d) Distribution of participants by World Bank income classification.

As with gender representation, the FSBI has made conscious efforts to improve international representation, both in its membership, members of council and attendees at its conferences. The Society preferably uses its FSBI acronym over the ‘Fisheries Society of the British Isles’ in communications to avoid obscuring its international scope and to avoid negative connotations associated with the term ‘British Isles’. In 2013, the Society also changed its logo to include the text ‘an international society for fish biology’. Beyond branding and communication, the Society offers multiple funding streams, all of which are available to its international membership, including travel, training and small research grants. In early 2020, the Society launched a Postdoctoral International Travelling Fellowship, specifically targeted at internationalisation of the Society. The FSBI also has long‐standing partnerships with the American Fisheries Society (AFS) and Japanese Society of Fisheries Science (JSFS), with two further international cooperation agreements with the Iberian Society of Ichthyology (SIBIC) and the Korean Society for Fisheries and Aquatic Sciences (KOSFAS) signed in 2025, not to mention the international standing of its journal, the *Journal of Fish Biology*. Despite improved internationalisation, future progress can still be made by increasing the diversity of representation, but crucially, also facilitating greater involvement of lower‐income countries, particularly in Africa.

To complement long‐term representation data with fine‐scale behavioural evidence, we recorded live talk durations for 68 presenters at the joint FSBI‐IFM 2025 symposium in Belfast, Northern Ireland. Allocated talk lengths were 10 min, including 2 min for questions. Speaking time was measured as the duration from the first spoken word to verbal conclusion, to the nearest second, excluding questions. Overtime was set to zero for anyone finishing in under 8 min or to the number of seconds over 8 min for anyone finishing after 8 min. Speaker gender was assessed as above, and career stage (junior: MSc or PhD student, senior: postdoctoral and beyond) was inferred from self‐identification, titles or online searches. Although overall gender representation among recorded speakers was balanced (48% women, 52% men), senior men represented a larger fraction of speakers (32%) than any other gender–career group among recorded speakers.

Overall, 78% of speakers exceeded their allocated time, with a mean overtime of 93 s (range: 3–212 s). When a 1‐min grace period was applied, this proportion dropped to 48.5%. Career stage significantly predicted the probability of exceeding the allotted time (binomial glm, χ^2^_1_ =5.15, *p* = 0.02, Figure 1b), whereas gender and the gender by career stage interaction effects were not supported (*p* > 0.05). Senior scientists had significantly higher odds of exceeding the allotted time (86%) than junior scientists (68%). This effect was consistent even after applying a grace period of up to 55 s (Figure S1). Overtime did not differ significantly between male and female speakers (Wilcoxon's *W* = 586, *p* > 0.05). However, career stage influenced overtime, with senior scientists spending significantly more time (ca. 30 s) beyond their allocation than junior scientists (Wilcoxon's *W* = 410, *p* = 0.043, Figure 1c).

These results align with some studies reporting that career stage – rather than gender per se – predicts timekeeping behaviour. In Edlund et al. (2018), allocated time, career stage and the level of timekeeping enforcement determined how long a presenter spoke. Käfer et al. (2018) did not find differences between men and women in excess time during their talks at the Society for Molecular Biology and Evolution (SMBE) conferences, but they did between faculty and students. Senior speakers may feel less constrained by formal limits, extending their total time. Alternatively, unconscious bias in audience behaviour or chair enforcement (potentially also affected by biases) could subtly reinforce senior scientists' greater visibility and tolerance for overtimes. Nonetheless, consistent overtime reduces discussion time and disadvantages later speakers, which cumulatively affects equity in exposure.

Other studies have shown that disparities can arise from geography and that racial and ethnic minoritized individuals remain underrepresented as speakers (Le et al., 2021; Wiley et al., 2024). Although some disparities could be assessed in our study, identifying multiple intersecting axes of identity (e.g., gender, ethnic background, LGBTQ+ identity) is both methodologically challenging and ethically contentious. This constrains our ability to quantify associated biases and to detect interactions driven by intersectionality, especially under limited sample sizes. Collecting self‐reported data on these characteristics could improve analyses and inform targeted actions, but it raises ethical and privacy concerns that make such collection sensitive.

Although the overtime dataset is modest and limited to one event, with gender only representing one dimension of diversity within the community, it demonstrates a simple, reproducible approach to quantify visibility dynamics in professional meetings. Continuing data gathering across fisheries conferences could reveal whether these patterns generalise and whether consistent moderation or timing policies reduce overruns.

We recommend that organisations systematically monitor and report speaker and membership demographics, as well as data on timekeeping, to aid representation, transparency and fairness. Individuals should also actively nominate, request and support colleagues from historically underrepresented backgrounds (Wiley et al., 2024). Interventions such as visible countdown timers, automatic microphone cutoff, strict moderation using cues and gender‐balanced session chairs have been shown to promote more equitable participation (Klein et al., 2017). For example, including women, early‐career researchers and other underrepresented groups on planning committees can dramatically reduce biases in representation (Casadevall & Handelsman, 2014).

## AUTHOR CONTRIBUTIONS

**Joshka Kaufmann:** conceptualization, data curation, analysis, visualisation and writing – original draft. **William Bernard Perry:** data curation, visualisation and writing – review and editing. Both authors have read, revised and agreed to the published version of the manuscript.

## Supporting information


**Figure S1.** *p*‐Values for career, gender and interaction effects from a generalised linear model (GLM) on overtime probability, depending on the threshold defining overtime in seconds.

## Data Availability

The data that support the findings of this study are openly available in zenodo at https://doi.org/10.5281/zenodo.18094385.
