# Supplementary material for: Reaching gender parity and improving internationalisation after five decades of FSBI symposia, but subtle career‐stage effects on timekeeping remain
Source: J Fish Biol. 2026 Apr 7;109(1):610–4. doi: 10.1111/jfb.70449 (PMC13397277; doi:10.1111/jfb.70449)
Supplement: Supplementary file 1 — Figure S1. p‐Values for career, gender and interaction effects from a generalised linear model (GLM) on overtime probability, depending on the threshold defining overtime in seconds. [file JFB-109-610-s001.docx]

Supplementary material for Kaufmann and Perry

**Reaching gender parity after five decades of FSBI symposia but subtle career-stage effects on timekeeping remain**


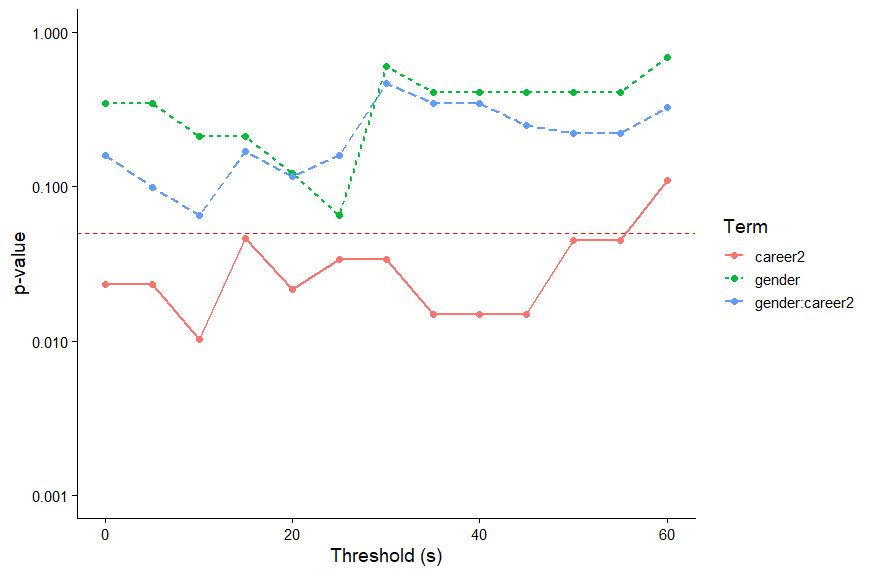


Figure S1 : P-values for career, gender and interaction effects on a GLM on overtime probability depending on threshold defining overtime in seconds.
